# A novel MPPT technology based on dung beetle optimization algorithm for PV systems under complex partial shade conditions

**DOI:** 10.1038/s41598-024-57268-8

**Published:** 2024-03-18

**Authors:** Chunliang Mai, Lixin Zhang, Xuewei Chao, Xue Hu, Xiaozhao Wei, Jing Li

**Affiliations:** 1 College of Mechanical and Electrical Engineering, Shihezi University, Shihezi, 832003 China; 2 Bingtuan Energy Development Institute, Shihezi University, Shihezi, 832000 China; 3 Engineering Training Center, Xinjiang University, Ürümqi, 830017 China

**Keywords:** Global maximum power point, Multiple peaks, Partial shading, Photovoltaic arrays, Dung beetle optimization algorithm, Energy harvesting, Renewable energy, Electrical and electronic engineering, Energy science and technology, Engineering

## Abstract

Solar power is a renewable energy source, and its efficient development and utilization are important for achieving global carbon neutrality. However, partial shading conditions cause the output of PV systems to exhibit nonlinear and multipeak characteristics, resulting in a loss of output power. In this paper, we propose a novel Maximum Power Point Tracking (MPPT) technique for PV systems based on the Dung Beetle Optimization Algorithm (DBO) to maximize the output power of PV systems under various weather conditions. We performed a performance comparison analysis of the DBO technique with existing renowned MPPT techniques such as Squirrel Search Algorithm, Cuckoo search Optimization, Horse Herd Optimization Algorithm, Particle Swarm Optimization, Adaptive Factorized Particle Swarm Algorithm and Gray Wolf Optimization Hybrid Nelder-mead. The experimental validation is carried out on the HIL + RCP physical platform, which fully demonstrates the advantages of the DBO technique in terms of tracking speed and accuracy. The results show that the proposed DBO achieves 99.99% global maximum power point (GMPP) tracking efficiency, as well as a maximum improvement of 80% in convergence rate stabilization rate, and a maximum improvement of 8% in average power. A faster, more efficient and robust GMPP tracking performance is a significant contribution of the DBO controller.

## Introduction

The world is facing emerging problems such as climate change, the depletion of traditional fossil fuels, and sustainable development. Solar power generation has become one of the most rapidly developed and largest industries in the renewable energy sector due to its green, clean, safe, and stable characteristics^1^. However, solar photovoltaic (PV) panels are very sensitive and susceptible to factors such as light intensity, temperature, and load. These dynamic behaviours make maximum power point tracking (MPPT) of PV arrays a challenge. In natural environments, PV arrays are shaded by trees, clouds, etc., resulting in uneven light and thus partial shading. Under resulting partial shading conditions (PSCs), the P-U characteristic curve of a PV array exhibits multiple peaks, including multiple local minima (LM) and a unique global minima (GM), which results in PV modules not being able to reach the optimal operating point at all times^2,3^. Therefore, effective MPPT for PV systems in complex environments is essential for improving solar energy utilization.

The MPPT of a PV system is mainly used to ensure that the output power is always stabilized at the maximum power point (MPP) by continuously adjusting the operating point of the PV modules, to increase the power generation output^4^. According to the output characteristics of the PV system, the current MPPT algorithms can be divided into three main categories^5^: (1) optimization model-based control algorithms; (2) control algorithms based on perturbation self-seeking optimization; and (3) control algorithms based on intelligent principles. Optimization model-based control algorithms mainly include open-circuit voltage detection^6^ and short-circuit current detection^7^. These types of methods are characterized by controlling the output voltage of the photovoltaic cell to ensure that it stabilizes near the set point value, which is set close to the maximum power point voltage under normal conditions. The control algorithm based on the optimization model has a simple structure, is easy to implement, has favourable stability, and reaches the set value faster. However, such methods cannot guarantee that the system truly operates at the maximum power point, and the system cannot be tracked in real time, which causes power loss when the external environment changes^8^. Control algorithms based on disturbance self-optimization mainly include Perturbation and Observation (P&O)^9,10^, and the Incremental Conductance Algorithm (ICA)^11,12^. These control algorithms directly measure the voltage and current output values of a PV power generation system and realize MPPT according to its output information without detecting changes in the external environment, and the structure is relatively simple. These control algorithms directly measure the voltage and current output of the PV power generation system and realize MPPT according to this output information, (without detecting changes in the external environment), and the corresponding structure is relatively simple. However, this type of algorithm exhibits poor convergence, steady state oscillations, and slow tracking speeds, leading to high energy loss. Simultaneously, this type of method fails when there are localized shadow conditions and changing irradiance^13^. To improve the MPPT tracking performance of this class of methods, researchers have improved them. Reference^14^ proposed an optimized hill-climbing method with dynamic step size, which eliminates the output steady-state oscillations. Reference^15^ improved the perturbation observation of hair by initiating a reverse strategy, which greatly improved the MPPT tracking performance of the traditional perturbation observation method. Reference^16^ proposes a 'reduced and fixed' improved perturbation observation method that reduces the output oscillations to zero and improves the response rate. However, the response speed of the system may decrease when the weather conditions are constantly changing. Control algorithms based on intelligent principles include fuzzy logic control (FLC)^17,18^, artificial neural network (ANN) control methods^19,20^, evolutionary computation (EC)^21–23^, and metaheuristic methods^24^. Reference^25^ used FLC control to realize multipeak MPPT. FLC has excellent convergence speed, but its practical use is affected by the experience of engineers. Reference^26^ used an ANN to realize multipeak MPPT, but the ANN needs a very specific and large amount of data to be trained to produce accurate results, and the application of the ANN has high hardware costs. Reference^27,28^ used a genetic algorithm and differential evolutionary algorithm to achieve steady-state tracking of MPPT, but this type of evolutionary algorithm exhibits poor tracking speeds. Metaheuristic algorithms, which are inspired by nature and human intelligence, offer great advantages in dealing with problems such as nonlinear optimization problems and thus have gradually become a hot research topic for researchers. Reference^29–31^ proposed three improved flower pollination algorithms and conducted performance tests under changing weather conditions, which showed that the improved algorithms substantially improved the tracking speed and tracking efficiency. Reference^32^ proposed a grid-connected PV MPPT technique using an arithmetic optimization algorithm to optimize the parameters of the proportional-integral (PI) controller, but the introduction of the PI controller increased the complexity of the system. Reference^33^ proposed an improved multistep constant current MPPT technique based on the grey wolf optimization algorithm and accurately obtained the maximum power point under different PSC conditions. Reference^34^ proposed an MPPT technique based on the Squirrel Search Algorithm (SSA) and simulated and experimentally analyzed the proposed algorithm under local shadowing conditions. Reference^35^ proposed a direct search cuckoo search (CS) MPPT technique with hardware-in-the-loop experimental validation. Reference^36^ proposed an MPPT technique based on the Horse Herd Optimization Algorithm (HOA) and experimentally validated it on a real PV system. Similar approaches include the Marine Predator Optimization Algorithm (MPA)^37^, Salp Swarm Algorithm (SSA)^38^, Search and Rescue Algorithm (SRA)^39^, and Tuna Swarm Optimization (TSO)^40^. The MPPT technique based on the Particle Swarm Optimization (PSO) algorithm^41^ is considered one of the more classical techniques, but the original Particle Swarm Optimization technique is slow in tracking and easily falls into local optimal solutions. Therefore, researchers have developed a series of enhanced particle swarm techniques. These include the adaptive factor selection particle swarm algorithm (FMSPSO)^42^, enhanced autonomous group particle swarm algorithm (EAGPSO)^43^, hybrid tandem particle swarm optimization algorithm (SSPSO)^44^, and hybrid particle swarm optimization with Salp Swarm Algorithm (PSOSSO)^45^. These improvement strategies improve the convergence performance of PSO to a certain extent. Moreover, to fully utilize the advantages of different algorithms, researchers have also proposed MPPT techniques based on hybrid algorithms, which are used to improve the tracking accuracy and speed. For example, reference^46^ hybridized the direct search technique Nelder-Mead with GWO and proposed a hybrid grey wolf algorithm (GWO-NM), which reduces unnecessary searches of particles and steady-state oscillations. Reference^47^ introduced the opposing reinforcement learning method into the butterfly optimization algorithm and proposed the hybrid butterfly optimization algorithm (OBRL-BOA), which reduces the oscillations caused by load changes. Reference^48^ designed a control model (SSA-PSO-DSMC) that mixes the Salp Swarm Algorithm with the particle swarm algorithm and combines the mixed algorithm with an intelligent direct-slip membrane controller; the of this model was verified on hardware-in-the-loop (HIL) systems. Similar hybrid MPPT techniques include Bird Swarm Fusion Skyhawk Optimization (BSFAO)^49^, Grey Wolf Optimization fused with Variable Step Incremental Conductance (GWO-VINC)^50^, Genetic Algorithm Hybrid Ant Colony Optimization Algorithm (GA-ACO)^51^, Improved Sparrow search algorithm with a hybrid adaptive neuro-fuzzy inference system (MSSA-ANFIS)^52^, and the hybrid crow search and pattern search (HCS-PS)^53^. Hybrid techniques are able to utilize the advantages of different algorithms and obtain satisfactory results, but this class of MPPT technique increases the complexity of the controller and increases the time cost. In addition, many of the above studies are limited to simulation studies, ignoring the experimental validation aspect.

Although many MPPT techniques based on metaheuristic algorithms have emerged, according to the ‘no free lunch’ principle, no algorithm can solve all optimization problems. The Dung Beetle Optimization (DBO) algorithm is a new population intelligence optimization algorithm proposed in 2022^54^, that is inspired by the natural behaviours of dung beetles such as rolling, foraging, stealing, and reproducing. Each of its four subpopulations performs four different searches with fixed population ratios, allowing different update rules to be sufficiently balanced between local and global searches. DBO has been applied to solve complex optimization problems in various fields^55,56^. According to the literature, there is a lack of simulation and experimental studies on the MPPT technique based on the DBO algorithm. Therefore, in this study, the maximum power tracking performance of the DBO technique under various weather conditions is investigated via simulation and experimental evaluation, and the results are compared and analyzed with those of other renowned MPPT techniques. The main contributions of this study are as follows:

1. A new MPPT technology is introduced for dealing with PS problems in PV systems. The population categorization and boundary selection strategy mechanisms in the DBO enable better GM global exploration and local convergence performance.
2. The proposed DBO approach solves the problems of long convergence time and settling time, abrupt power oscillations and poor adaptability of other metaheuristic technologies. In addition, it can reach the GM with fewer iterations while guaranteeing zero power oscillation at the GM.
3. Combined with actual field atmospheric data, the effectiveness of DBO in practical applications is further verified.
4. Based on the hardware experimental analysis of the HIL + RCP platform, the DBO is also able to effectively track the GM.

The remainder of this paper is organized as follows. Section "PV modelling and the effect of shading conditions on PV arrays", describes the topology of the PV system and its output characteristics under PSCs. Section "DBO-based MPPT technique", describes the dung beetle optimization algorithm and its MPPT implementation for PV systems. Section "Results and discussion", describes the MATLAB/Simulink-based simulation results in detail, including a comparative study with other advanced metaheuristic algorithms for the MPPT technique. Section "Experimental verification", reports the experimental results obtained using the physical platform HIL + RCP. Finally, Section "Conclusion", provides the conclusions and future directions of this study.

## PV modelling and the effect of shading conditions on PV arrays

### Modelling of PV cells

**Figure 1:**
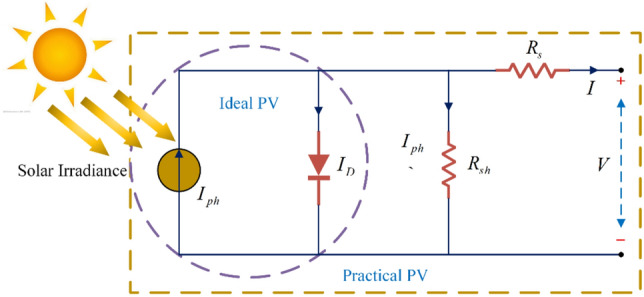
Single-diode PV cell model.

PV cells utilize the photovoltaic effect to convert solar energy into electrical energy. A single-diode model is used to simulate the PV cell as shown in Fig. 1. The single-diode model neglects complex losses in the depletion region^57^. The model simplifies the PV cell into a current source, a diode, a parallel resistor R_sh_ and a series resistor R_s_. where the current source is mainly affected by the irradiance. The ideal diode model is represented by the purple dashed line, while a fixed resistance is added to the diode model used in practice. I_ph_ represents the current generated by the irradiation, and I_D_ denotes the current through the diode. Applying Kirchhoff's current law, the output current of the photovoltaic cell is calculated by Eq. (1):

1 $$I = I_{ph} - I_{o} \left\{ {\exp \left[ {\frac{{q\left( {V + R_{s} I} \right)}}{AkT}} \right] - 1} \right\} - \frac{{V + R_{s} I}}{{R_{sh} }}$$

where V and I are the output voltage and output current of the PV cell, respectively; I_o_ is the equivalent diode reverse saturation current; q is the electronic charge constant, which is 1.6 × 10^−19^ C; k is Boltzmann's constant, which is 1.38 × 10^−23^ J/K; A is the diode quality factor; and T is the cell operating temperature.

2 $$I_{ph} = \frac{G}{{G_{STC} }}\left[ {I_{sc\_STC} + k_{i} \left( {T - T_{STC} } \right)} \right]$$

3 $$I_{0\_STC} = \frac{{I_{sc\_STC} }}{{\exp \left( {\left( {qV_{oc\_STC} /AKT_{STC} } \right) - 1} \right)}}$$

where I_sc_STC_ denotes the short-circuit current of the PV cell under standard test conditions; G_STC_ = 1000 W/m^2^, T_STC_ = 25 °C; G denotes irradiance; k_i_ denotes the short-circuit current coefficient; E_g_ denotes the energy bandgap of the semiconductor; and V_oc_STC_ denotes the open-circuit voltage of the PV cell under reference conditions.

The output voltage, current and power of a single solar cell are small and cannot meet the normal operating requirements, so photovoltaic modules are usually manufactured from multiple solar cells connected in series and parallel. The output characteristic equation of the PV module with N_m_ connections in series and N_p_ connections in parallel is modified as Eq. (5). R_s_eq_ denotes the equivalent series resistance and R_p_eq_ denotes the equivalent parallel resistance. The PV module model used in this study is "LSP672-285" and its electrical characteristics are listed in Table 1 and described as follows:

4 $$I = N_{p} I_{ph} - N_{p} I_{o} \left\{ {\exp \left[ {\frac{{q\left( {V + R_{s\_eq} I} \right)}}{{N_{m} AkT}}} \right] - 1} \right\} - \frac{{V + IR_{s\_eq} }}{{R_{p\_eq} }}$$

**Table 1:** Characteristic parameters of photovoltaic modules.

PV module parameter	Symbol	LSP672-285
Maximum power	P_max_	285 W
Current at P_MPP_	I_MPP_	7.92 A
Voltage at P_MPP_	V_MPP_	36 V
Open circuit current	V_OC_	44.5 V
Short circuit current	I_SC_	8.53 A
Temperature coefficients of V_OC_	K_V_	− 0.32%/°C
Temperature coefficients of I_SC_	K_I_	0.02%/°C

### PV module under partial shadow conditions

**Figure 2:**
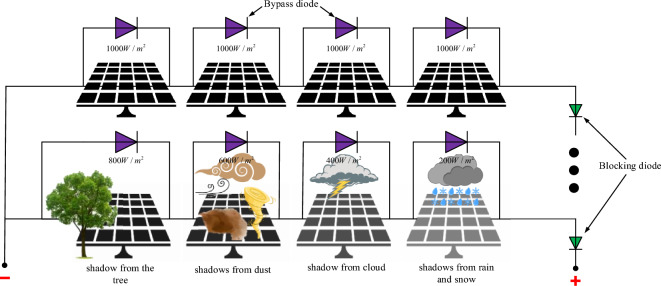
Series connected PV modules under uniform and non-uniform irradiance.

**Figure 3:**
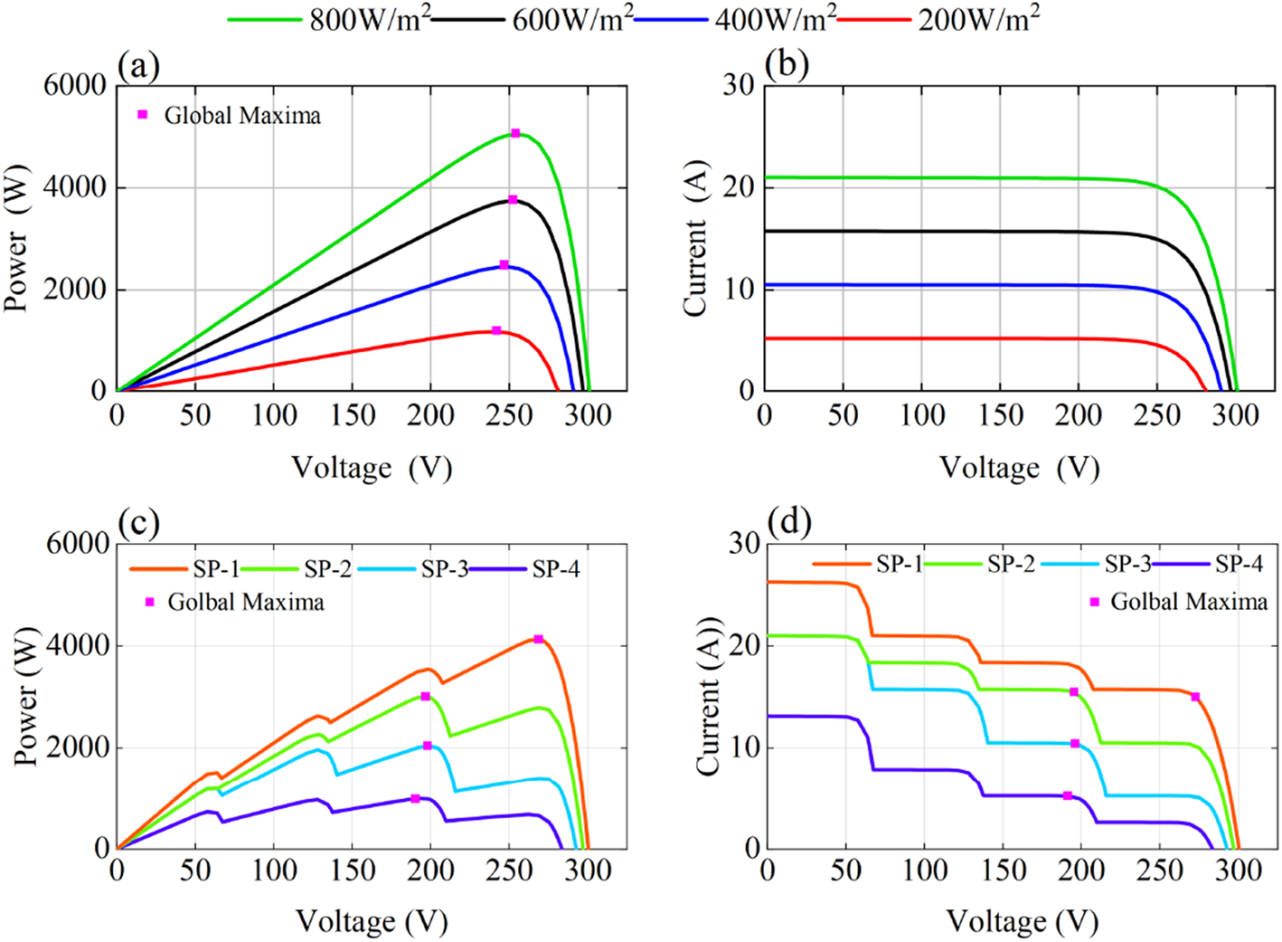
(**a**) P–V curve under uniform irradiance, (**b**) I–V curve under uniform irradiance, (**c**) P–V curve under non-uniform irradiance and (**d**) I–V curve under non-uniform irradiance.

In the field, a PV array may be intermittently shaded by clouds, buildings, trees, dust, etc., as shown in Fig. 2. At these times, the output characteristics of the shaded PV cells change, leading to output power loss and hot spot effects, and the PV array efficiency decreases. Thus, the output characteristics of the PV array exhibit multipeak characteristics. As shown in Fig. 3a, b, under uniform irradiation, the P–V and I–V curves of the PV arrays have only one peak, namely, GMPP. However, under nonuniform irradiation, there is one GM and multiple LMs. As shown in Fig. 3c, d.

### Design of boost converters

**Figure 4:**
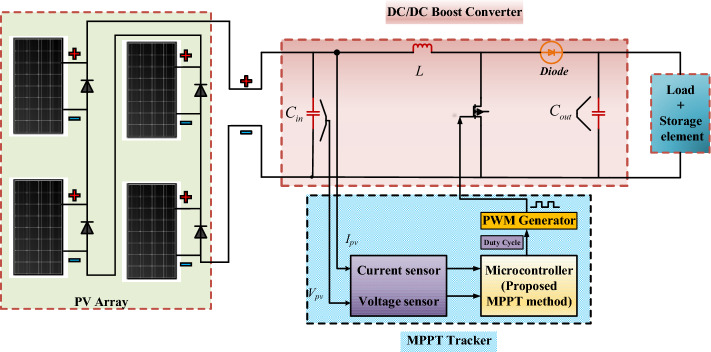
Typical PV system with MPPT control.

The topology of the MPPT control system of a typical PV system is shown in Fig. 4. The DC–DC boost converter connects the PV panel to the back-end load or energy storage unit. The voltage of the PV array is regulated by adjusting the duty cycle, the main control variable of the converter, as a way of obtaining the best reference voltage V_refh_ for MPPT control of the PV system. The duty cycle has a value between 0 and 1. The parameters of the DC boost converter are the input voltage V_DCin_, output voltage V_DCout_, inductor L, input capacitance C_in_, output capacitance C_out,_ switching frequency f_swt_, and duty cycle D. Among these parameters, the input capacitance is used to reduce the ripple generated by the photovoltaic module, and the input ripple voltage ΔV_load_ = 1%V_DCin_. The role of the output capacitor is to limit the ripple of the output voltage, and the output ripple voltage is ΔV_load_ = 2%V_DCout_. The abovementioned parameters are calculated using Eqs. (4)–(8). The output voltage and current of the PV module are influenced mainly by the load and the operating environment of the PV module.

5 $$V_{DCout} = \frac{{V_{DCin} }}{{1 - D_{cycle} }}$$

6 $$D_{cycle} = \frac{{t_{on} }}{{t_{swt} }}$$

7 $$C_{in} = \frac{{D_{cycle} }}{{8 \times f_{swt} \times L \times 0.01}}$$

8 $$L = \frac{{D_{cycle} \times \left( {1 - D_{cycle} } \right)^{2} \times r}}{{2 \times f_{swt} }}$$

9 $$C_{out} = \frac{{D_{cycle} }}{{0.02 \times f_{swt} \times r}}$$

## DBO-based MPPT technique

Inspired by the rolling, dancing, foraging, stealing and breeding behaviours of dung beetles, we propose a new MPPT technology for PV systems based on the DBO algorithm. In the DBO algorithm, the global search refers to the rolling ball behaviour, e.g., rolling the manure ball to the optimal position for storage; Local searches (including breeding, foraging, and stealing) are then used to move around the storage balls to further explore the optimal location. The working process of the dung beetle MPPT controller is as follows: initialize the dung beetle population, evaluate the fitness values of different populations and record the optimal individuals. Then, each subpopulation was subjected to collaborative optimization. A global search was performed with the Rolling Dung Beetle and the Dancing Dung Beetle. The local search was conducted by breeding and foraging dung beetles, and the spawning and foraging areas were dynamically adjusted with the change in R parameters to reduce the search range. Thief dung beetles search for the best foraging areas to speed up tracking.

### Inspiration

The Dung beetle optimizer (DBO) is a new biological swarm intelligence optimization algorithm proposed by Jiankai in 2022. The algorithm is inspired by the social behaviour of dung beetle populations and is designed with five different update rules to help find high-quality solutions. The population classification and boundary selection strategies of DBO enable the algorithm to consider both global exploration and local convergence.

### Implementation

#### Ball-rolling dung beetle

A dung beetle makes a ball of dung and rolls it to a desired location. During the rolling process, the dung beetle needs to keep the dung ball rolling in a straight line by using celestial cues (sun position or wind direction, etc.). To simulate the rolling behaviour, the dung beetle needs to move in a given direction throughout the search space. The position update formula for rolling dung beetles is as follows:

10 $$\begin{aligned} & x_{i} \left( {t + 1} \right) = x_{i} \left( t \right) + \alpha \times k \times x_{i} \left( {t - 1} \right) + b \times \Delta x, \\ & \Delta x = \left| {x_{i} \left( t \right) - X^{w} } \right| \\ \end{aligned}$$

where t is the current number of iterations; x_i_(t) is the position information of the ith dung beetle at t iterations; k ∈ (0,0.2] is a constant value representing the coefficient of deflection; b is a constant between (0, 1); and α is a natural coefficient assigned to − 1 or 1, with 1 denoting no deviation and − 1 denoting a deviation from the original direction. X^W^ is the global worst position, and Δx is used to simulate light intensity variation.

When a dung beetle encounters an obstacle that prevents it from moving forwards, it changes the direction of travel by dancing. The tangent function is used to simulate the dancing behaviour of the dung beetle for a new rolling direction. The location of the Dancing Dung Beetle is thus updated as follows:

11 $$x_{i} \left( {t + 1} \right) = x_{i} \left( t \right) + \tan \left( \theta \right)\left| {x_{i} \left( t \right) - x_{i} \left( {t - 1} \right)} \right|$$

where θ ∈ [0,π]. If θ is equal to 0 or π/2, the position of the dung beetle will not be updated.

#### Breeding dung beetles

Certain dung balls are hidden in a relatively safe environment as an egg-laying place. Therefore, a boundary selection strategy is used to model the area where female dung beetles lay their eggs:

12 $$\begin{aligned} & Lb* = \max \left( {X^{*} \times \left( {1 - R} \right),Lb} \right), \\ & Ub* = \min \left( {X^{*} \times \left( {1 + R} \right),Ub} \right) \\ \end{aligned}$$

where X^*^ represents the current local optimal position, Lb^*^ is the lower bound of the spawning area, Ub^*^ is the upper bound, R = 1 − t∕Tmax, Tmax is the maximum number of iterations, and the upper and lower bounds of the optimization problem are represented by Lb and Ub, respectively.

Female dung beetles lay their eggs in the spawning area, and each female dung beetle produces only one egg in each iteration. The boundary of the spawning area is determined by the value of R, which dynamically changes. The position of the hatching ball is also dynamic during the iteration process, and the iteration process is as follows:

13 $$B_{i} \left( {t + 1} \right) = X^{*} + b_{1} \times \left( {B_{i} \left( t \right) - Lb^{*} } \right) + b_{2} \times \left( {B_{i} \left( t \right) - Ub^{*} } \right)$$

where B_i_(t) is the position information of the ith hatching ball at the tth iteration. b_1_ and b_2_ represent two independent 1 × D random vectors, and D denotes the dimension of the optimization problem. The location of hatching balls is strictly limited to the spawning area.

**Figure 5:**
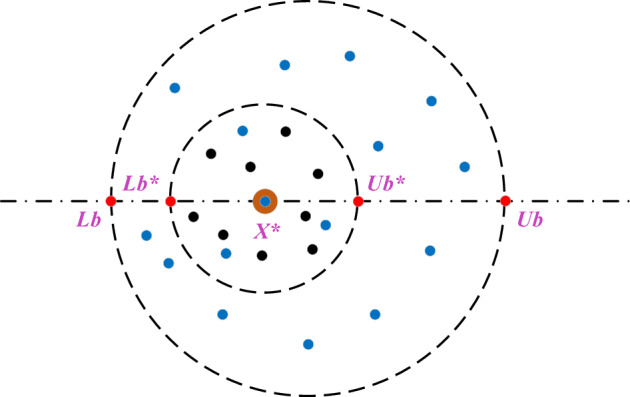
Boundary selection strategy.

The boundary selection strategy is shown in Fig. 5. The brown circle indicates the current local optimal position X^*^, and the rolling dung beetle is indicated by the blue point. Small black points indicate hatching balls, each of which contains only one egg. The upper and lower bounds of the boundary are indicated by red circles.

#### Small dung beetles (foraging dung beetles)

Eggs that hatch successfully become baby dung beetles. Dung beetles are guided to forage by establishing optimal foraging areas. The boundaries of the optimal foraging area are delineated as follows:

14 $$\begin{aligned} & Lb^{b} = \max \left( {X^{b} \times \left( {1 - R} \right),Lb} \right) \\ & Ub^{b} = \min \left( {X^{b} \times \left( {1 + R} \right),Ub} \right) \\ \end{aligned}$$

where X^b^ denotes the global optimal position. The lower and upper bounds of the optimal foraging area are represented by Lb^b^ and Ub^b^, respectively. The position of the small dung beetle is updated as follows:

15 $$x_{i} \left( {t + 1} \right) = x_{i} \left( t \right) + C_{1} \times \left( {x_{i} \left( t \right) - Lb^{b} } \right) + C_{2} \times \left( {x_{i} \left( t \right) - Ub^{b} } \right)$$

where x_i_(t) denotes the location information of the ith small dung beetle at the tth iteration, C_1_ denotes a random number that follows a normal distribution, and C_2_ denotes a random vector belonging to (0,1).

#### Stealing dung beetles

Certain dung beetles steal dung balls from other dung beetles, a process that is denoted as stealing dung beetles. During the corresponding iterations, the position of the thief is updated as follows:

16 $$x_{i} \left( {t + 1} \right) = X^{b} + S \times g \times \left( {\left| {x_{i} \left( t \right) - X^{*} } \right| + \left| {x_{i} \left( t \right) - X^{b} } \right|} \right)$$

where x_i_ (t) is the location information of the ith thieving dung beetle at the tth iteration, g is a random vector of size 1 × D obeying a normal distribution, and S is a constant value.

### Implementation of DBO-based MPPT technology under PS conditions

**Figure 6:**
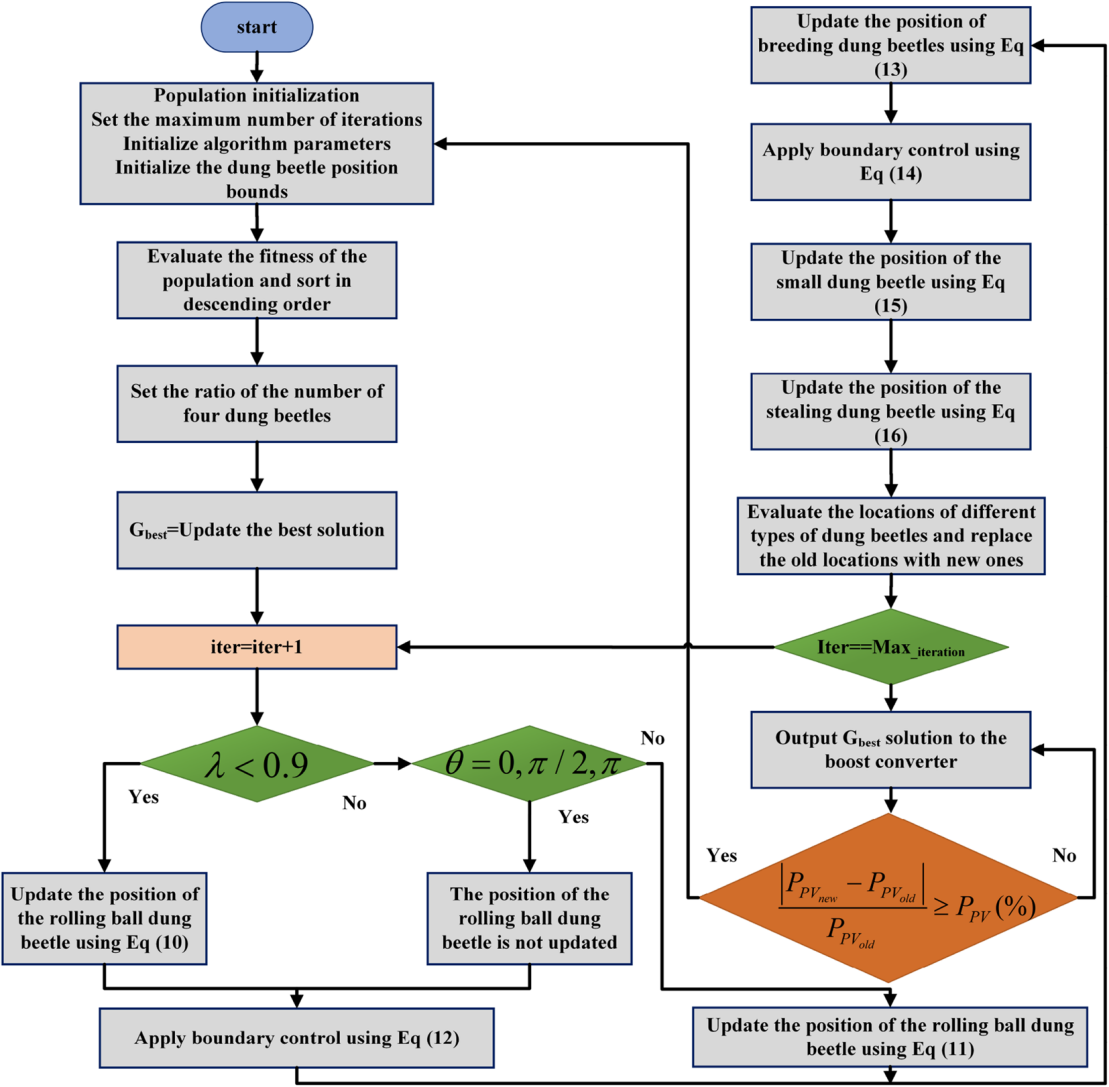
Flow chart of MPPT control based on the DBO algorithm.

The implementation of DBO-based MPPT technology is shown in Fig. 6. The proposed DBO control algorithm is used to adjust the duty cycle of the PV power generation system to maximize the output power of the PV system and reduce energy loss. Therefore, the objective function (OF) is defined as shown in Eq. (17). P_PV_ denotes the output power of the PV array, and the duty cycle is varied from 0 to 1. The current position of an individual dung beetle indicates the duty cycle. DBO attempts different duty cycle values based on the real-time current and voltage parameters of the PV array and evaluates the fitness value corresponding to each duty cycle until it finds the optimal duty cycle corresponding to the maximum value of the output power.

17 $$OF = Max\left( {P_{PV} } \right)$$

When the operating temperature or irradiance of the PV array changes, the position of the MPP changes accordingly. Therefore, a restart strategy^44,45^ is introduced in the algorithm to ensure its adaptability to dynamically changing weather. The algorithm is initialized with the following conditions:

18 $$if\frac{{\left| {P_{{PV_{new} }} - P_{{PV_{old} }} } \right|}}{{P_{{PV_{old} }} }} \ge P_{PV} \left( \% \right)$$

In the DBO, rational classification of dung beetle populations, effective boundary selection strategies, and reinitialization produce effective results. The initialization of DBO particles and multiple particle updating methods increase the speed of the process.

The pseudocode for the implementation of the MPPT technique based on the dung beetle optimization algorithm is shown in Table 2.

**Table 2:**
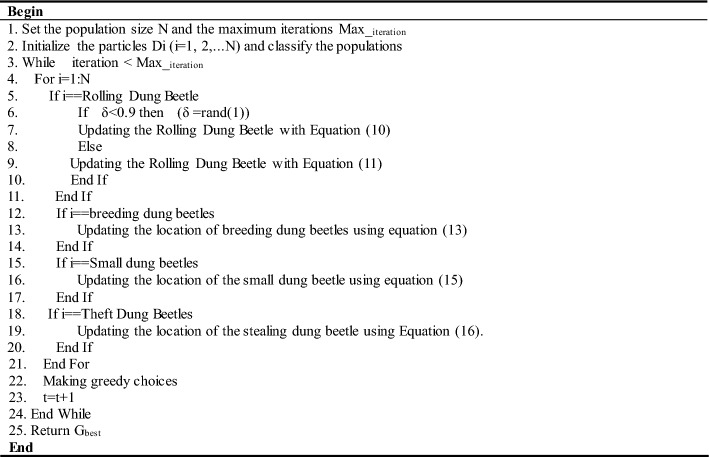
Pseudocode of the MPPT technique based on the DBO algorithm.

## Results and discussion

An MPPT simulation model of the PV system is designed in MATLAB/Simulink 2022b and comprises a PV array (the PV array is in a 5 × 1 series configuration, where each PV module is in a 2 × 3 configuration), a boost converter, a resistive load, and an MPPT controller. The detailed parameters of the PV modules used are listed in Table 1. The parameters of the boost converter are set as C_in_ = 200 µF, C_out_ = 500 µF, and L = 8.5 mH. The IGBT switching frequency is set to 20 kHz and the load is 20 Ω.

In this section, Case 1 is a static PSC, Case 2 is a dynamic PSC, Case 3 is a CPS, and Case 4 contains field atmospheric data used to evaluate the real-time performance of the DBO-based MPPT technology at 24 h. To verify the various performances of the DBO algorithm, several classical and recent MPPT techniques, such as the SSA^34^, CS^35^, HOA^36^, PSO^41^, FMSPSO^42^, and GWO-NM^46^, are introduced for comparative analysis. The initialization parameters are set to be the same for all algorithms. Based on these results, a comprehensive performance evaluation of DBO with other technologies was performed.

### Partial shading condition: Case-1

**Figure 7:**
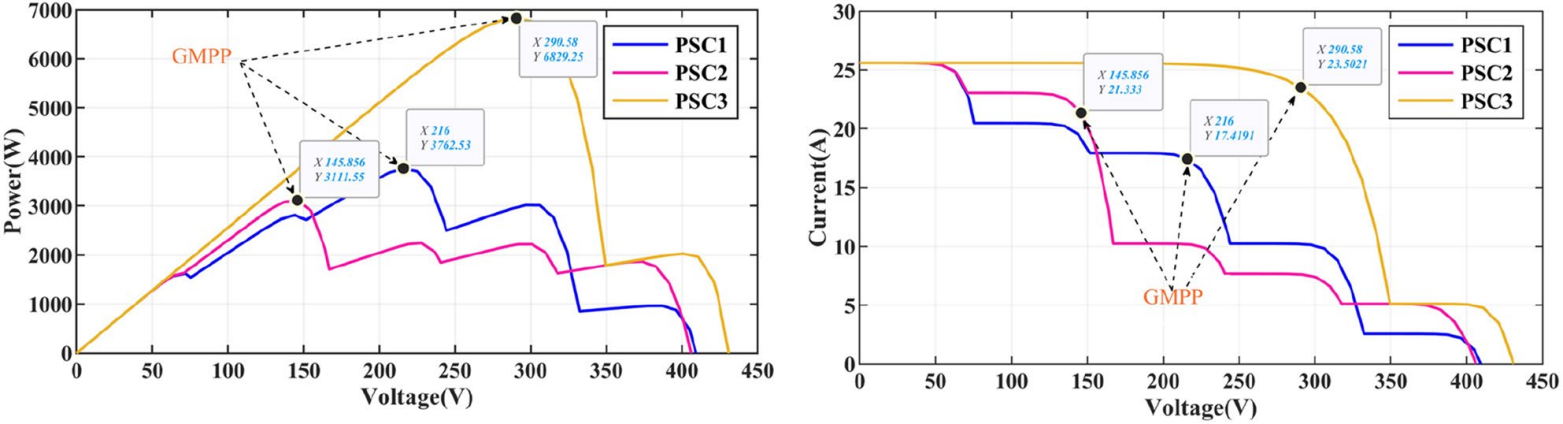
P–V and I–V curves of PV array under Case-1.

The irradiance pattern of the PV array under Case-1 is shown in Table 3. The environmental temperature is 25 °C. GMPP under three different PSCs is located on the left, centre, and right of the curve, as shown in Fig. 7.

**Table 3:** Irradiance patterns of five PV arrays.

Shade condition	Irradiances (W/m^2^)					MPP (W)	Location (GMMP)
1. PSC_1_	100	400	700	800	1000	3762.5	Middle
2. PSC_2_	200	300	400	900	1000	3111.6	Left
3. PSC_3_	1000	1000	1000	1000	200	6829.3	Right
4. PSC_1_–PSC_3_–PSC_2_						3762.5–6829.3–3111.6	Middle–right–left

**Figure 8:**
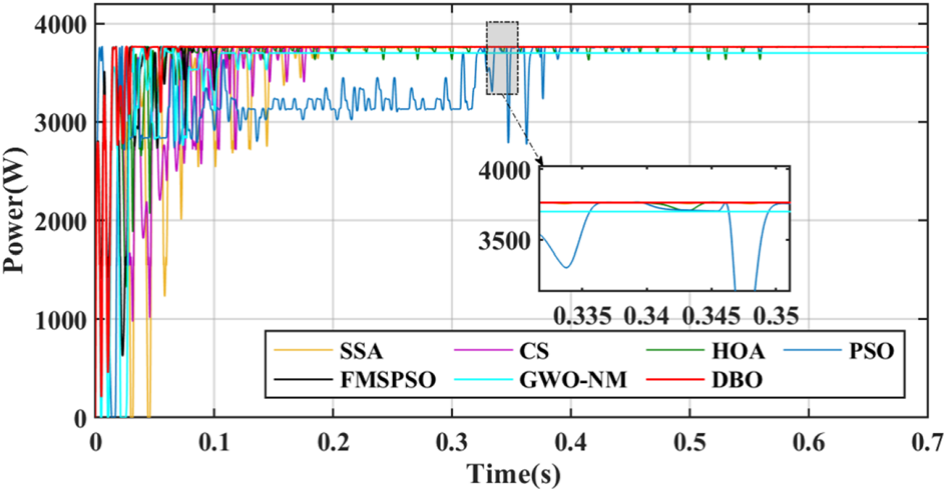
Power comparison in PSC1.

Under PSC1, the GM of the PV system is 3762.5 W. The power transient curve is shown in Fig. 8. The PSO tracks the GM for the longest time because the PSO fell into the LM at 0.07 s and tried to attract all the particles to the LM until 0.323 s retreated to the GM and jumped out of the LM trap. The tracking speed of GWO-NM is 0.149 s, which is closer to that of DBO. However, the final maximum power obtained by GWO-NM is only 3698.98 W, which is lower than that of the other algorithms. The distinction between GM and LM in PSC1 is high, and all seven technologies can handle PS problems. The maximum powers tracked by DBO, SSA, CS, HOA, PSO, FMSPSO and GWO-NM are 3762.4 W, 3760.1 W, 3761.9 W, 3762.0 W, 3758.3.7 W, 3759.6 W and 3698.98 W respectively, and the tracking efficiencies of GM reach 99.99%, 99.93%, 99.98%, 99.91%, 99.88%, 99.92% and 98.31%, respectively. In contrast, the tracking accuracy and tracking efficiency of DBO are better than those of the other six technologies.

In terms of tracking speed, the convergence time, interval time and settling time are used to evaluate the global exploration and local convergence ability of MPPT technology. The interval time is the difference between the settling time and the convergence time. The convergence times of DBO, SSA, CS, HOA, PSO, FMSPSO and GWO-NM are 0.017 s, 0.065 s, 0.087 s, 0.044 s, 0.023 s, 0.029 s and 0.032 s, the settling times are 0.084 s, 0.354 s, 0.237 s, 0.565 s, 0.452 s, 0.105 s, and 0.149 s, and the interval times are 0.057 s, 0.289 s, 0.150 s, 0.521 s, 0.577 s, 0.076 s and 0.117 s respectively. The time consumption of the DBO algorithm is the lowest and remains within 0.1 s, indicating that it can consider both global and local exploration in the search process. While other technologies may exhibit shorter convergence times, they typically require longer local convergence times. This process causes a loss of power and reduces the energy output of the PV module.

**Figure 9:**
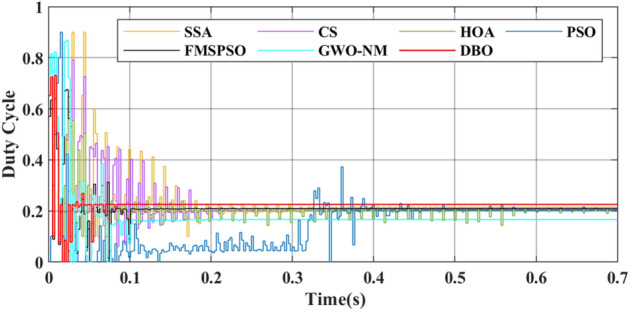
Duty cycle comparison in PSC1.

For metaheuristic algorithms based on MPPT technology, the number of iterations of the algorithm when it reaches stability is another important indicator for measuring the performance of the algorithm. A comparison of the duty cycles of the different technologies is shown in Fig. 9. The DBO can track the GM after 5 iterations, and the response is fast. The SSA, CS, HOA, PSO, FMSPSO and GWO-NM require more than 20 iterations to track the GM.

**Figure 10:**
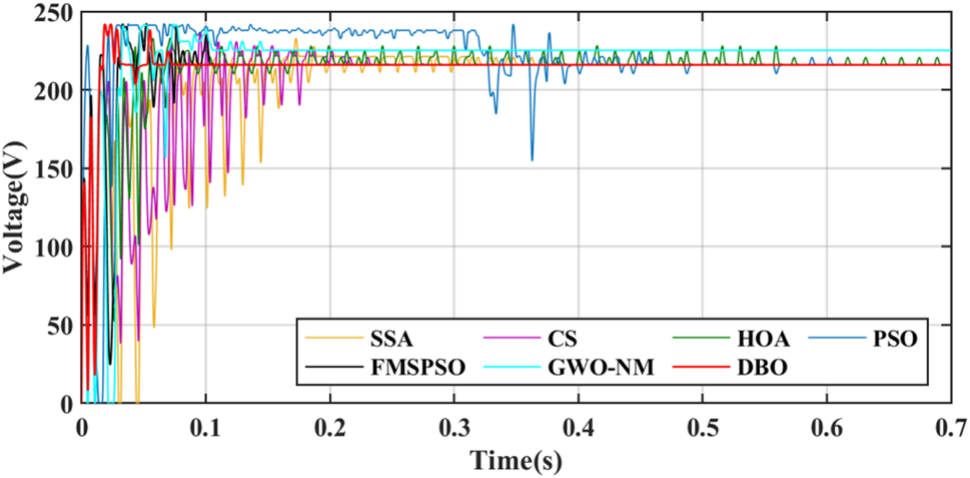
Voltage comparison in PSC1.

GM power oscillations and fluctuations cause energy losses, especially in the HOA and PSO. The amplitude of the power fluctuations of the HOA and PSO are between 9 and 30 W because the speed vector update formulas for the PSO and HOA are embedded with random numbers. Irregular oscillations and fluctuations in voltage are also observed in the transient voltage comparison in Fig. 10.

**Figure 11:**
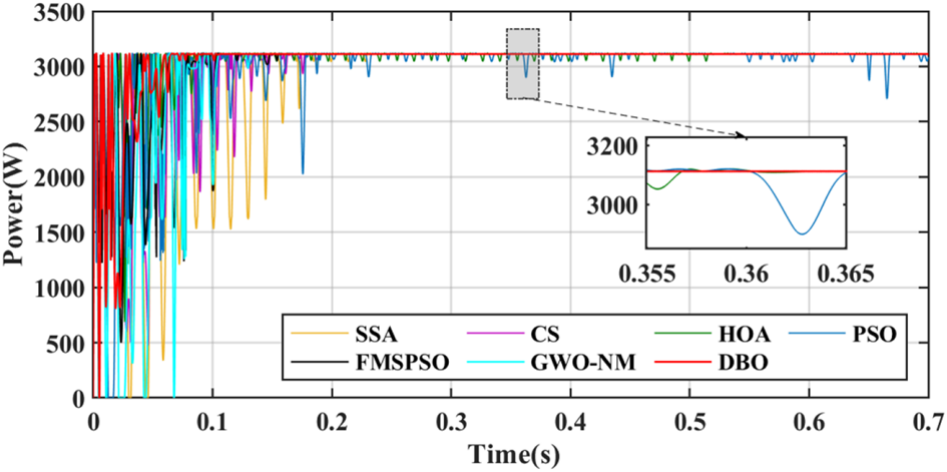
Power comparison in PSC2.

The output characteristic curve of PV cells under PSC2 has 5 knee points, and the power values of the 3 knee points on the right are relatively close to each other, which challenges efficient operation of the MPPT. The corresponding power curve is shown in Fig. 11. The convergence times of DBO, SSA, CS, HOA, PSO, FMSPSO and GWO-NM are 0.030 s, 0.052 s, 0.049 s, 0.032 s, 0.063 s, 0.032 s and 0.051 s respectively. Their settling times are 0.109 s, 0.243 s, 0.208 s, 0.523 s, 0.147 s, 0.159 s and 0.134 s respectively. The power curve at PSC2 is slightly more complex than that at PSC1. The highest power output of PSC2 is obtained by DBO at 3111.5 W, followed by CS at 3111.4 W, GWO-NM at 3111.2 W, SSA at 3111.1 W, FMSPSO at 3110.8 W, HOA at 3110.1 W, and PSO at 3082.8 W. The tracking efficiencies of DBO, SSA, CS, HOA, PSO, FMSPSO and GWO-NM are 99.99%, 99.98%, 99.99%, 99.96%, 99.07%, 99.97% and 99.98%, respectively.

**Figure 12:**
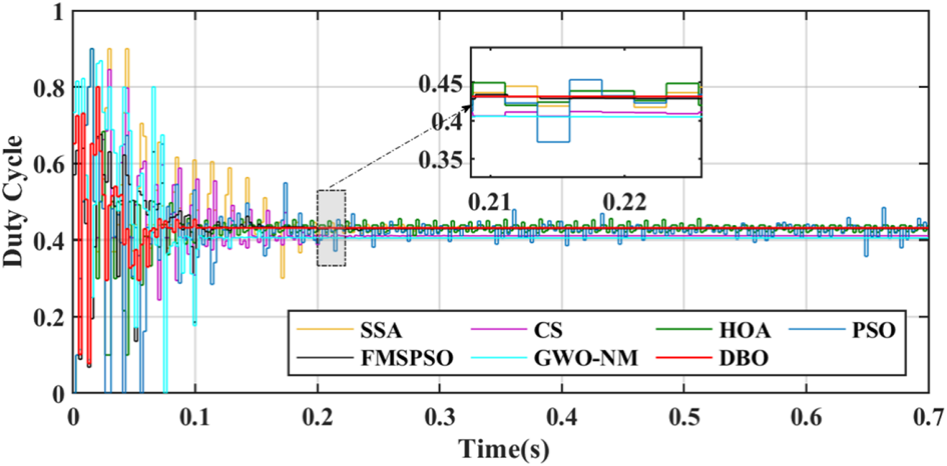
Duty cycle comparison in PSC2.

**Figure 13:**
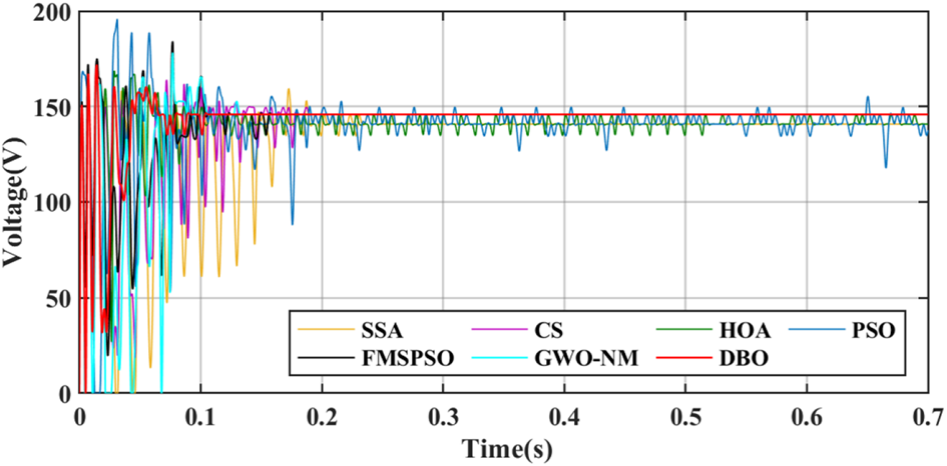
Voltage comparison in PSC2.

A comparison of the duty cycles under PSC2 is shown in Fig. 12. The number of iterations required for DBO is 8, while other MPPT technologies require more than 30 iterations. The voltage comparison is shown in Fig. 13, with the highest voltage obtained by the DBO being 145.8 V. Obvious voltage fluctuations at the GM still appear in the HOA and PSO, and the duty cycle cannot be stable. The corresponding power curve also has power fluctuations of up to 45 W, and the energy loss in this process is inevitable. The energies harvested by the DBO, SSA, CS, HOA, PSO, FMSPSO and GWO-NM were 60.66 kJ, 60.62 kJ, 60.51 kJ, 60.48 kJ, 60.41 kJ, 60.59 kJ and 60.63 kJ, respectively. The greatest energy gain at the DBO by the DBO derives from the shortest settling time and zero oscillation of power at the GM, and the lower energy harvests of the HOA and PSO are due to the longer tracking time and sustained oscillation of power at the GM.

### Rapidly changing shade conditions: Case-2

Case-2 is studied to examine the adaptability of the MPPT technology for dynamic PSCs. The PV array configuration and DC-DC converter parameters are the same as those in Case-1. The PSC sequence is PSC1-PSC3-PSC2, which is divided into three phases with an interval of 1 s. The details are shown in Table 3. The position of the GM changes from middle-right-left.

**Figure 14:**
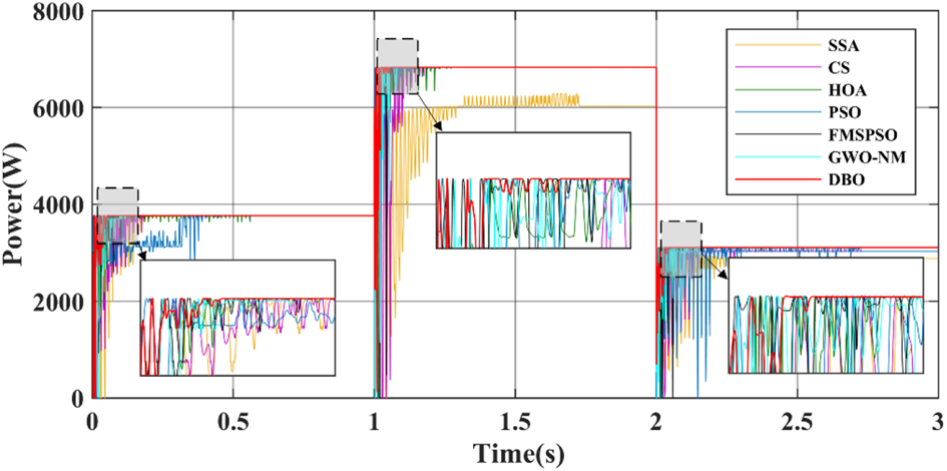
Power comparison in Case-2.

Figure 14 shows a power comparison under the Case 2. At 1 s, PSC1 changes to PSC3. PSC3 has a GM of 6829.3 W. The power tracked by DBO is 6829.2 W, and the power tracked by SSA, CS, HOA, PSO, FMSPSO and GWO-NM is 6023.4 W, 6829.1 W, 6828.5 W, 6829.0 W, 6828.1 W and 6828.3 W, respectively. The SSA cannot achieve secondary stable tracking of the GM, and the tracking power is the lowest. The other MPPT technologies enable secondary tracking of GM, and the tracking time of the DBO is the shortest.

At 2 s, PSC3 changes to PSC2. PSC2 has a GM of 3111.6 W. The powers of the DBO, SSA, CS, HOA, PSO, FMSPSO and GWO-NM tracks are 3111.5 W, 2885.2 W, 3037.5 W, 3109.6 W, 3037.7 W, 3107.2 W, and 3105.9 W, respectively. The SSA still cannot track GM after three restarts and falls into LM. The PSO tracked the wrong GM after continuous irregular oscillations. Compared with that of PSC2 in Case-1, the tracking efficiency of the DBO algorithm when it is dynamically cut into PSC2, which is 99.99%, remains unchanged. The tracking efficiencies of the SSA, CS, HOA, PSO, FMSPSO and GWO-NM decreased by 7.62%, 2.38%, 0.03%, 1.45%, 0.12% and 0.17%, respectively. It can be concluded that the DBO has good adaptability to dynamic PSCs and can quickly search and track two or three times according to changes in the external environmental irradiance. This approach shows good robustness in the multistage tracking process.

The average power of the 3 stages is shown in Table 4. The average power levels of DBO, SSA, CS, HOA, PSO, FMSPSO and GWO-NM are 4530 W, 4063 W, 4333 W, 4502 W, 4409 W, 4515 W, and 4500 W, respectively. DBO has the highest average power, while a higher average power signifies that more energy is harvested. Similarly, we analyze the average convergence time, average interval time, and average settling time. The average convergence times of DBO, SSA, CS, HOA, PSO, FMSPSO and GWO-NM in the 3 stages are 0.022 s, 0.119 s, 0.092 s, 0.037 s, 0.028 s, 0.037 s and 0.027 s, while the average settling times are 0.076 s, 0.454 s, 0.244 s, 0.411 s, 0.491 s, 0.139 s and 0.130 s, their intervals are 0.051s, 0.299 s, 0.152 s, 0.374 s, 0.463 s, 0.102 s and 0.103 s, respectively. DBO takes the shortest time, indicating that this technology can also track the GM efficiently and accurately under dynamic PSC. The newly proposed DBO technology has strong adaptability to dynamic PSCs and can improve the production efficiency of PV systems.

**Table 4:** Quantitative comparison of the DBO with the SSA, CS, HOA, PSO, FMSPSO and GWO-NM for Case-2.

Tech	Avg convergence time (s)	Avg interval time	Avg settling time (s)	Pavg at GM (W)	MPPavg tracked (W)	Avg power (W)	Energy (kJ)	Effie. (%)
DBO	0.022	0.051	0.076	4567.8	4567.7	4530	187.96	99.99
SSA	0.119	0.299	0.454	4567.8	4223.6	4063	166.28	92.46
CS	0.092	0.152	0.244	4567.8	4492.1	4333	179.06	98.33
HOA	0.037	0.374	0.411	4567.8	4567.5	4502	187.13	99.98
PSO	0.028	0.463	0.491	4567.8	4543.1	4409	184.43	99.45
FMSPSO	0.037	0.102	0.139	4567.8	4564.9	4515	186.82	99.93
GWO-NM	0.027	0.103	0.130	4567.8	4544.3	4500	186.15	99.48

**Figure 15:**
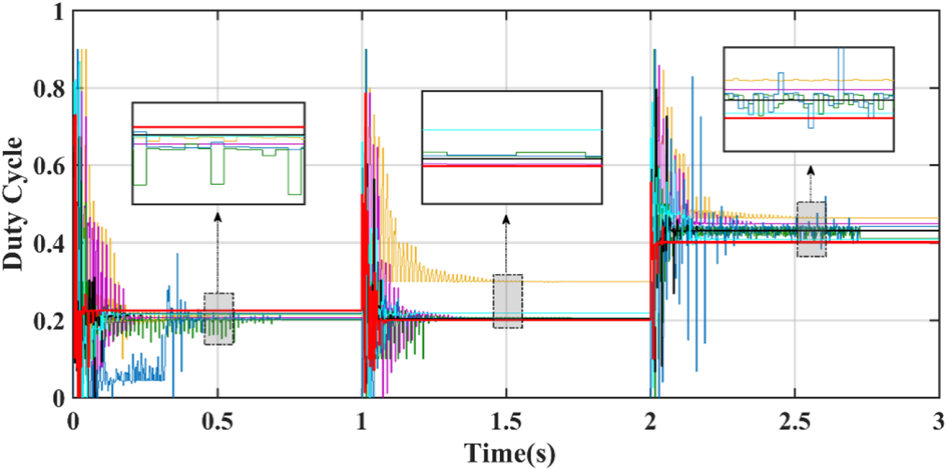
Duty cycle comparison in Case-2.

**Figure 16:**
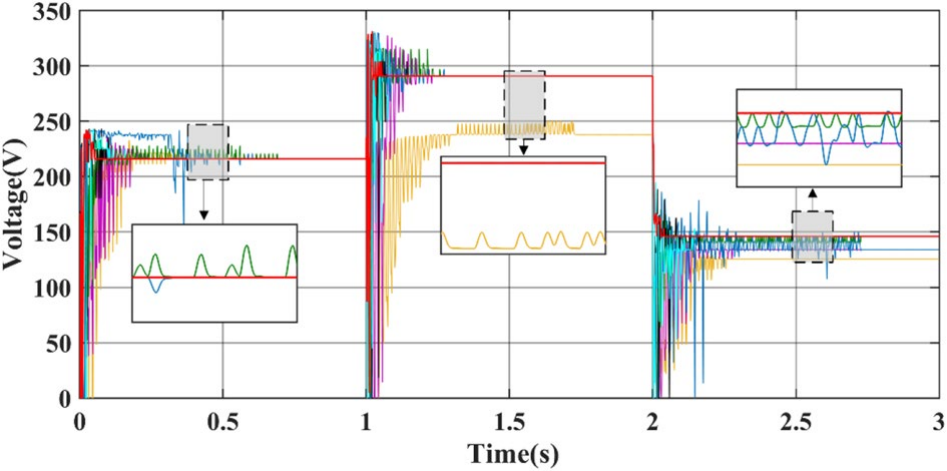
Voltage comparison in Case-2.

Figures 15 and 16 show the duty cycle and voltage comparison. Under dynamic PSC, the PSO and HOA still have large voltage oscillations at the GM, which is caused by the algorithm mechanism and is difficult to eliminate. The CS and SSA produce large oscillations at high frequencies during the global search due to the Levy flight mechanism in the algorithm. While this effect improves the algorithm’s ability to find the best duty cycle, it takes more time to execute tracking. FMSPSO sets multiple clusters and adds an adaptive selection factor strategy to PSO. In addition, the GWO-NM mixes the NM into GWO; these improved strategies improve the tracking speed and accuracy of the maximum power point to some extent, and the energy collection is further improved compared with the SSA, CS, HOA and PSO. However, it is still lower than the energy captured by DBO. Simultaneously, the introduction of improved strategies increases the complexity of the algorithm, increases the computational burden of the computer, and requires a longer computation time. DBO uses the population classification mechanism and boundary selection strategy to conduct collaborative optimization, which exhibits faster convergence speed and good robustness (because these are inherent features of the algorithm). In Case 2, the average number of iterations for DBO at GM was only 6, while the other approaches exceeded 20 iterations.

### Complex partial shading (CPS) condition: Case-3

**Figure 17:**
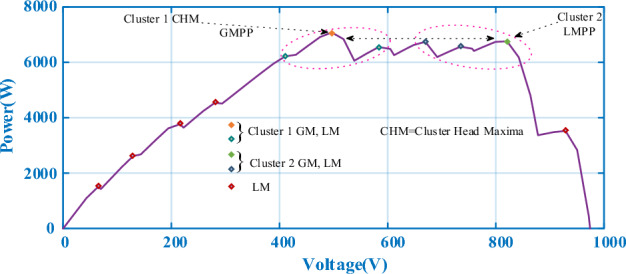
P–V characteristic curve at CPS.

The PV array in the photovoltaic power station is combined in the form of multiple panels in series/parallel, and when exposed to nonuniform irradiance, a complex peak form of multiple LMs will be produced, which can be called a CPS. The PV array adopts a 12 × 1 series configuration, and the specific irradiance parameters of the different panels are shown in Table 5. The parameters of the DC-DC converter are the same as those in Case-1. The P–V output curve of the PV array under a CPS is shown in Fig. 17. There are three extreme points in Cluster 1, from left to right, and their corresponding maximum powers are 6216.8 W at 410.4 V, 7078.7 W at 496.8 V, and 6552.8 W at 583.2 V. There are also three extreme points in Cluster 2, and the maximum power values from left to right are 6744.6 W at 669.5 V, 6566.2 W at 734.4 V, and 6762.4 W at 820.8 V. The CHM of 7078.7 W in Cluster 1 was GMPP, and the CHM in Cluster 2 was 6744.4 W.

**Table 5:** Irradiance pattern of Case-3.

Case	Irradiance $\left( {\frac{{\text{W}}}{{{\text{m}}^{2} }}} \right)$			P_max_ (W)
Case-2	PV1:330	PV5:570	PV9:400	7078.7
	PV2:640	PV6:440	PV10:150	
	PV3:800	PV7:1000	PV11:600	
	PV4:730	PV8:350	PV12:600

**Figure 18:**
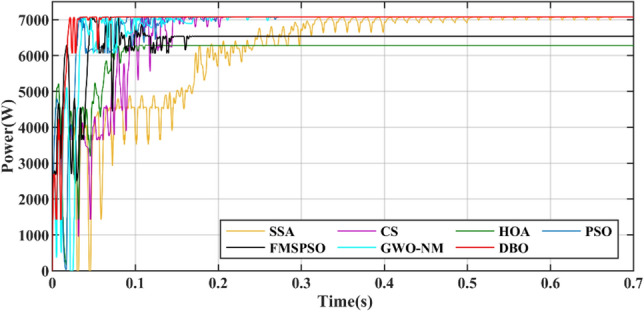
Power comparison in Case-3.

**Figure 19:**
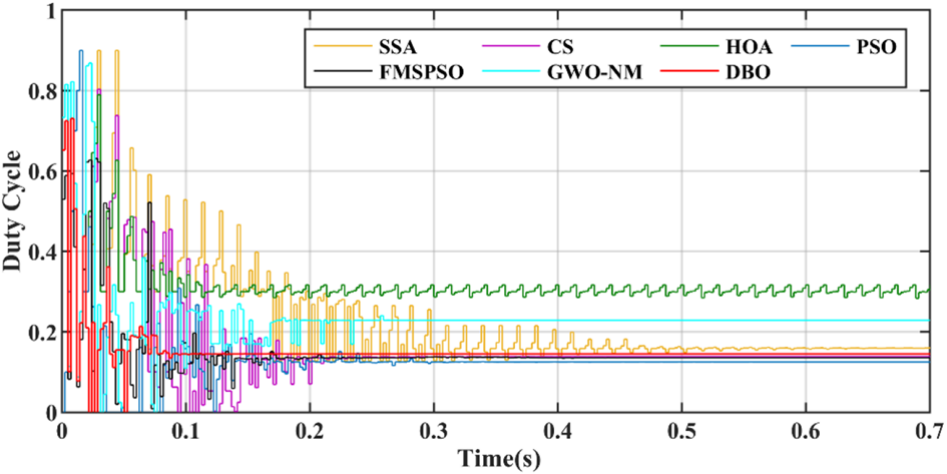
Duty cycle comparison in Case-3.

The power comparison under CPS is shown in Fig. 18. A detailed comparison of the duty cycle iteration behaviour is shown in Fig. 19. HOA and FMSPSO are trapped in the LM during the search process and remain stable at low power, and energy loss is inevitable. The SSA spends the longest time in the exploratory phase, despite leaving the LM after 1.5 s. CS, PSO and GWO-NM successfully avoided the LM trap, but ultimately did not find the ideal GM, which also caused some energy loss. DBO spends slightly more settling time in CPS than in PSC1, depending on the complexity of the irradiance.

**Figure 20:**
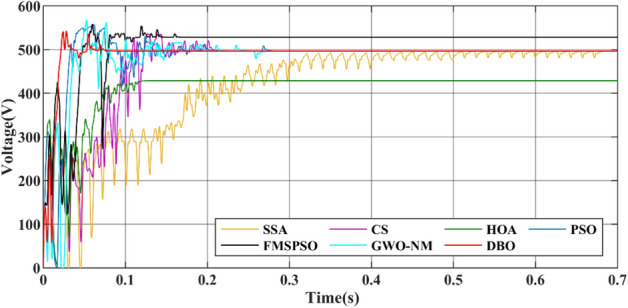
Voltage comparison in Case-3.

The convergence times of DBO, SSA, CS, HOA, PSO, FMSPSO and GWO-NM are 0.041 s, 0.322 s, 0.109 s, 0.081 s, 0.064 s, 0.048 s and 0.047 s, respectively, and the settling times are 0.091 s, 0.509 s, 0.105 s, 0.153 s, 0.209 s, 0.172 s and 0.266 s, respectively. In terms of tracking efficiency and accuracy, the maximum powers tracked by DBO, SSA, CS, HOA, PSO, FMSPSO and GWO-NM are 7078.4 W, 7077.1 W, 7077.3 W, 6281.3 W, 7075.2 W, 6542.74 W and 7076.53 W, respectively. DBO also has the highest tracking accuracy and efficiency. The DBO gradually stabilized after 6 iterations. The numbers of iterations required for the SSA, CS, HOA, PSO, FMSPSO and GWO-NM are 33, 15, 6, 30, 10 and 18, respectively. The response speed of the DBO is the most ideal. A voltage comparison under a CPS is shown in Fig. 20.

HOA has a tracking efficiency of more than 99% under PSC1 and PSC2, but under PSC3, its tracking efficiency is only 75.45%, producing minimal energy. FMSPSO suffers from the same problem, achieving more than 99% tracking efficiency under PSC1 and PSC2, but only 92.42% under PSC3. Due to the complexity of PSCs, the tracking effectiveness of different MPPT technologies is different, indicating that some MPPT technologies have poor adaptability to complex irradiance and can only solve relatively simple PS problems but cannot address CPSs. The newly proposed DBO technology shows good environmental adaptability in dealing with PS problems.

Table 6 lists the overall performance of the DBO controller under PSC1, PSC2, and the CPS. The tracking efficiency of the DBO is almost close to 100%. Simultaneously, due to its efficient GM tracking and fast convergence performance, the DBO algorithm is able to capture more equal energy under various PSCs.

**Table 6:** Quantitative comparison of the DBO with SSA, CS, HOA, PSO, FMSPSO and GWO-NM.

Tech	Psc No	Convergence time (s)	Interval time	Settling time (s)	Power at GM (W)	Power tracked (W)	Energy(kJ)	Effie. (%)
DBO	PSC1	0.017	0.057	0.084	3762.5	3762.4	73.36	99.99
	PSC2	0.030	0.079	0.109	3111.6	3111.5	60.66	99.99
	CPS	0.041	0.049	0.091	7078.7	7078.4	69.12	99.99
SSA	PSC1	0.065	0.289	0.354	3762.5	3760.1	73.29	99.93
	PSC2	0.052	0.191	0.243	3111.6	3111.1	60.62	99.98
	CPS	0.322	0.187	0.509	7078.7	7077.1	67.5	99.97
CS	PSC1	0.087	0.150	0.237	3762.5	3761.9	73.33	99.98
	PSC2	0.049	0.158	0.208	3111.6	3111.4	60.51	99.99
	CPS	0.109	0.105	0.214	7078.7	7077.3	69.021	99.98
HOA	PSC1	0.044	0.521	0.565	3762.5	3759.1	73.26	99.91
	PSC2	0.032	0.485	0.523	3111.6	3110.1	60.48	99.96
	CPS	0.081	0.071	0.153	7078.7	LM 5341.3	51.984	75.45
PSO	PSC1	0.023	0.577	0.452	3762.5	3758.3	71.49	99.88
	PSC2	0.063	0.147	0.210	3111.6	3082.8	60.41	99.07
	CPS	0.064	0.145	0.209	7078.7	7075.2	69.012	99.95
FMSPSO	PSC1	0.029	0.076	0.105	3762.5	3759.6	73.29	99.92
	PSC2	0.038	0.127	0.159	3111.6	3110.8	60.59	99.97
	CPS	0.048	0.124	0.172	7078.7	6542.74	56.85	92.42
GWO-NM	PSC1	0.032	0.117	0.149	3762.5	3698.98	72.14	98.31
	PSC2	0.051	0.083	0.134	3111.6	3111.2	60.63	99.98
	CPS	0.047	0.219	0.266	7078.7	7076.53	68.61	99.96

### Conditions change with weather: Case-4

**Figure 21:**
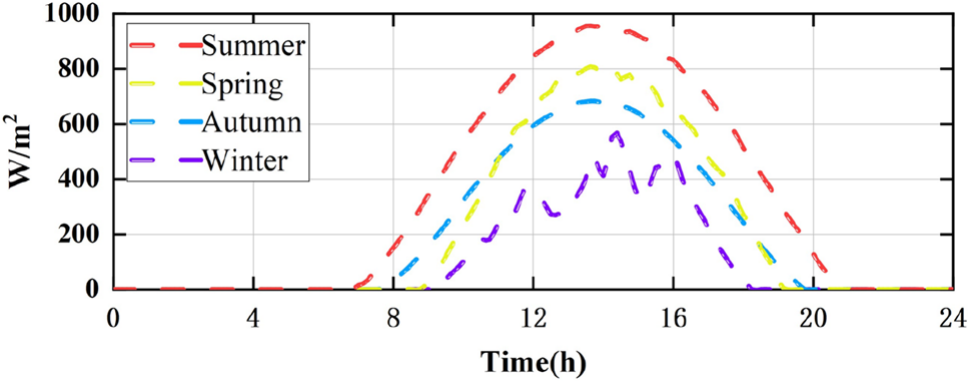
Irradiance levels in Shihezi in four seasons.

Cases 1–3 analyse static, dynamic, and short-term irradiance changes. Case-4 was combined with field atmospheric data from Shihezi city, Xinjiang, China, to study the performance of MPPT technology under long-term, dynamic PSCs. Field atmospheric data such as irradiance and temperature changes are provided by Xinjiang photovoltaic power stations. The irradiance levels of the four seasons in Shihezi city are shown in Fig. 21, and the irradiance and temperature data are real-time 24-h data. The PV system used is the same as that in Case-1. In this section, the 24-h peak power, average power and energy harvest of the PV system are used to verify the performance of MPPT technology.

**Figure 22:**
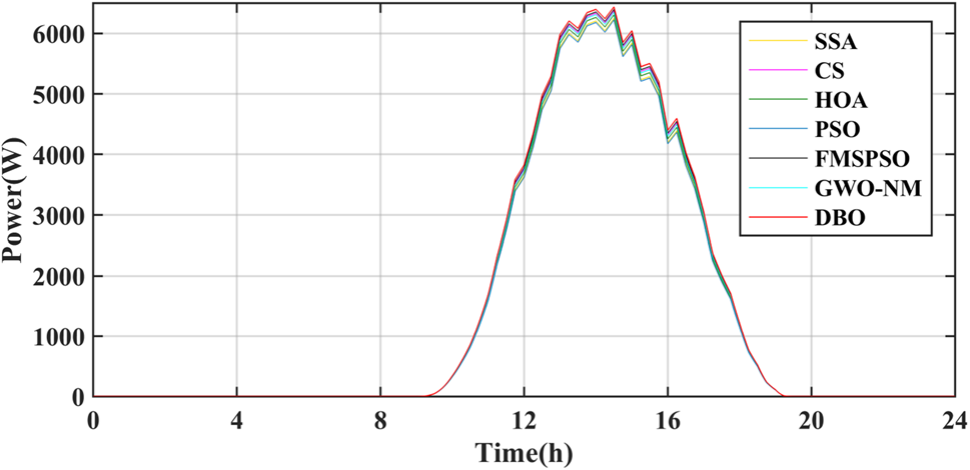
Power tracked in spring.

**Figure 23:**
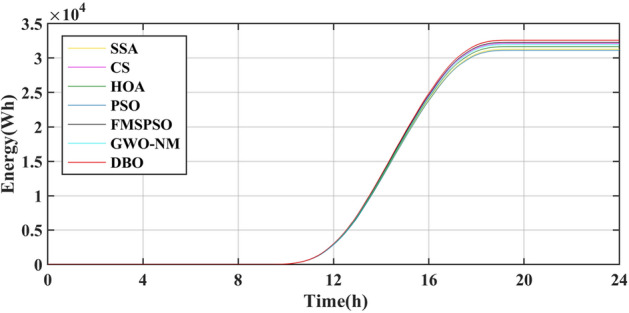
Energy harvested in spring.

The results of the different MPPT technologies are listed in Table 7. The DBO has more average and peak power in spring and summer, so it can harvest more energy. The MPPT technology based on DBO contributes to the global goal of zero carbon emissions. The tracked power and harvested energy of different MPPT technologies in the spring are shown in Figs. 22 and 23. The energy harvested by the DBO in 24 h is 32.59 KWh, the SAA is 31.66 KWh, the CS is 31.11 KWh, the HOA is 30.32 KWh, the PSO is 30.68 KWh, the FMSPSO is 32.28 KWh, and the GWO-NM is 31.96 KWh. The peak power of the DBO is 6440 W, followed by the SSA at 6309 W, the CS at 6223 W, the HOA at 6094 W, the PSO at 6154 W, the FMSPSO at 6398 W, and the GWO-NM at 6352 W; the average powers of these approaches are 1380 W, 1341 W, 1318 W, 1285 W, 1300 W, 1367 W, and 1354 W, respectively. The energy yield of DBO is approximately 5% greater than that of other technologies.

**Table 7:** Summary of Case 4 atmospheric data.

City	Season	Measurement	DBO	SSA	CS	HOA	PSO	FMSPSO	GWO-NM
Shihezi	Summer	Energy (kWh)	53.66	52.31	53.20	52.73	52.14	53.31	53.05
		Peak P (W)	8137	8099	8132	8118	8090	8135	8128
		Avg. P (W)	2329	2271	2310	2289	2264	2316	2303
	Spring	Energy (kWh)	32.59	31.66	31.11	30.32	30.68	32.28	31.96
		Peak P (W)	6440	6309	6223	6094	6154	6398	6352
		Avg. P (W)	1380	1341	1318	1285	1300	1367	1354

**Figure 24:**
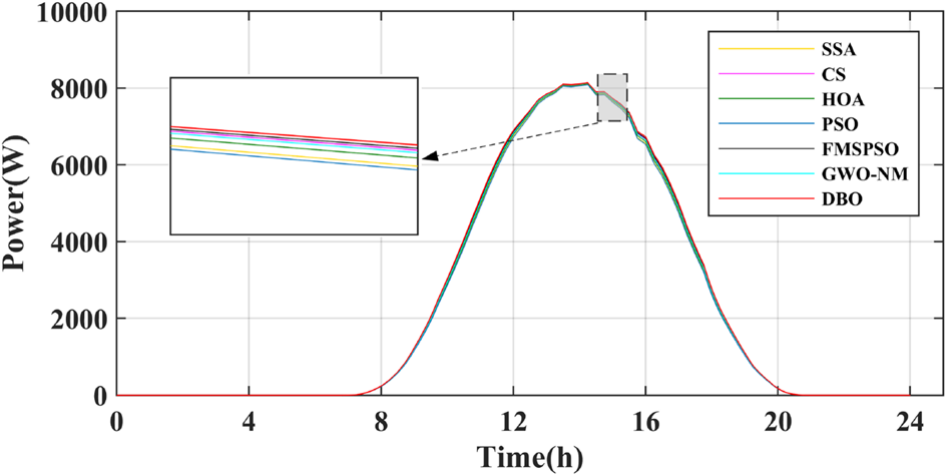
Power tracked in summer.

**Figure 25:**
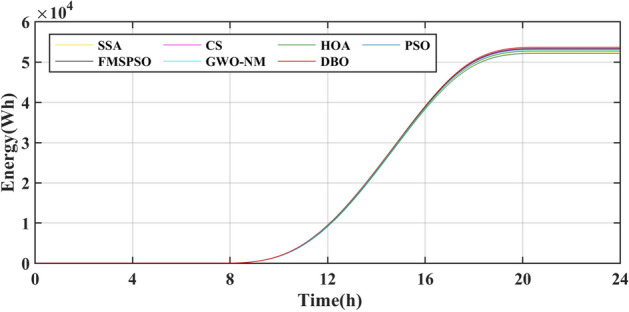
Energy harvested in summer.

Summer has the highest solar irradiance levels throughout the year, enabling more energy harvesting than in other seasons. The power and energy harvesting results are shown in Figs. 24 and 25. The energy harvested by the DBO in summer is 53.66 KWh, the SSA is 52.31 KWh, the CS is 53.20 KWh, the HOA is 82.73 KWh, the PSO is 52.14 KWh, the FMSPSO is 53.31 KWh, and the GWO-NM is 53.05 KWh. The peak power and average power of the DBO are 8137 W and 2329 W, the SSA is 8099 W and 2271 W, the CS is 8132 W and 2310 W, the HOA is 8118 W and 2289 W, the PSO is 8089 W and 2264 W, the FMSPSO is 8135 W and 2316 W, and the GWO-NM is 8128 W and 2303 W. Compared to other technologies, DBO has increased energy production by approximately 3% in the summer. DBO technology can adapt to practical application environments. The energy harvested by the PV system comes from two main sources. A small part of this energy is captured by the search process, while the majority of it is captured in the steady state. DBO has a faster convergence speed, accurate steady-state power, and no oscillations at GM, enabling it to minimize the energy loss in the search process and therefore capture more energy.

### Efficiency and performance assessment

**Figure 26:**
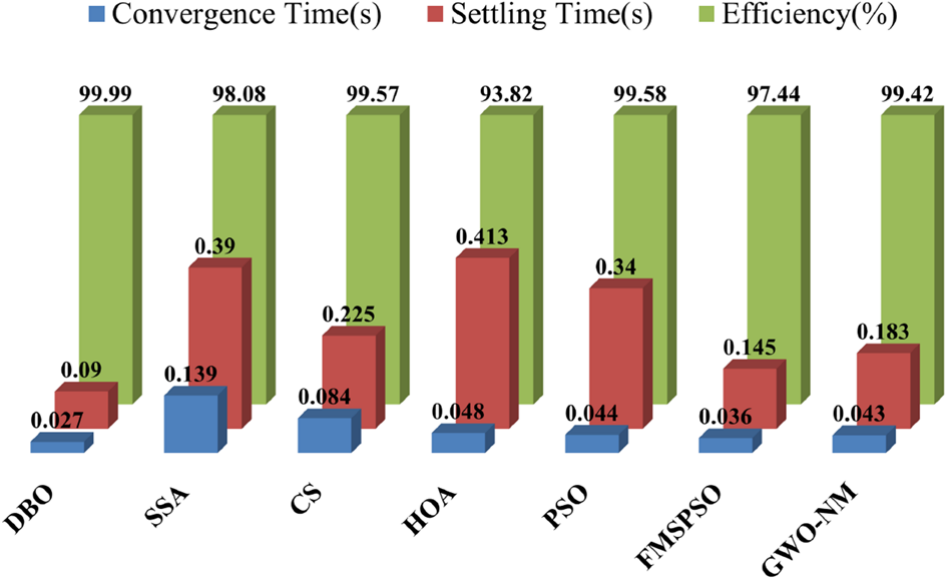
Average convergence time, settling time and tracking efficiency for different MPPT technologies.

**Figure 27:**
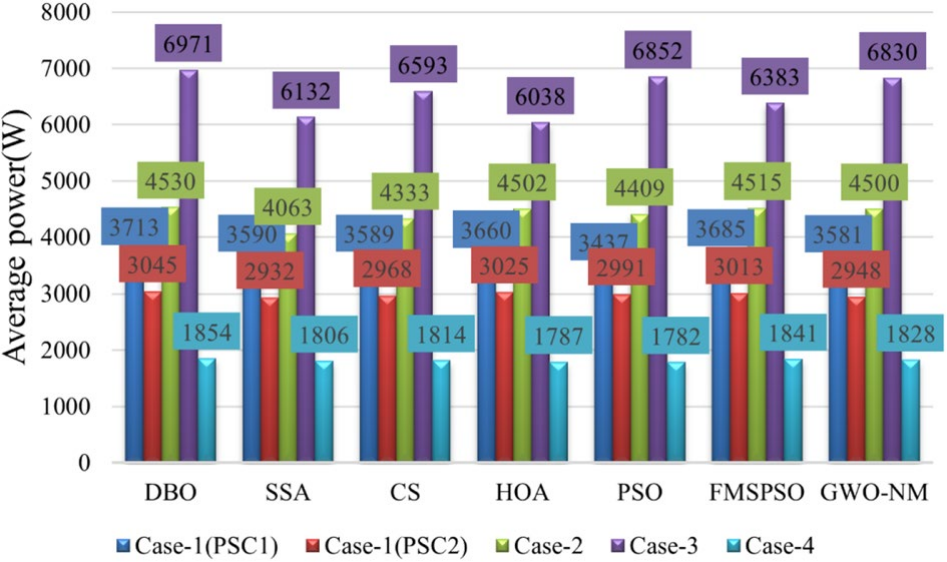
Comparison of average power statistics for different MPPT technologies.

Figures 26 and 27 show the results of the statistical quantitative analysis of the different technical performance indicators. Regardless of whether traditional P&O technology or bioinspired MPPT control technology is used, the most prominent problem is still the oscillation of the GM and the tendency to fall into a local optimal solution, which leads to a low power generation efficiency of the PV system. By testing the performance of DBO technology under various conditions, it can be found that the DBO output curve at the GM is smooth and almost oscillation-free, and the energy loss is reduced to a very low level. At the same time, DBO can track GM within approximately 30 ms (on average) and can reach stable GM within 90 ms. Compared with other MPPT technologies, the convergence rate is increased by 38–80%, and the stability rate is increased by 60–80%. The average power per unit of time reflects the energy harvesting ability of the MPPT technology, and there is a positive correlation between these values. The average power of the DBO is increased by 3–8%, and the harvested energy is similarly increased by the same percentage. Efficient global exploration and robust local exploitation enable DBO to converge quickly and quickly achieve stable GM tracking. The DBO tracking efficiency can reach 99.99% under all weather conditions, which allows it to capture more energy in complex environments. Moreover, the proposed MPPT technique is compared to other renowned and latest MPPT techniques using various criteria, as shown in Table 8. Where the average computation time is the average of the time consumed in computing PSC1, PSC2 and CPS. The DBO technique is less complex and requires less computational time than the other approaches.

**Table 8:** Comparative analysis of different MPPT techniques under various criteria.

MPPT algorithms	SSA^34^	CS^35^	HOA^36^	PSO^41^	FMSPSO^42^	GWO-NM^46^	Proposed
Periodic tunning	Not required	Not required	Not required	Not required	Not required	Not required	Not required
Tracking accuracy	High	High	High	Good	High	High	Very high
Steady-state oscillation	Zero	Zero	Minor	Minor	Zero	Zero	Zero
Tracking speed	Medium	Medium	Medium	Medium	Fast	Fast	Very fast
Algorithm complexity	Medium	Medium	Medium	Simple	More	More	Medium
Efficiency	High	Average	Average	Average	High	High	High
Average computational time	11.88 s	12.63 s	12.11 s	11.39 s	15.91 s	16.28 s	10.51 s

## Experimental verification

**Figure 28:**
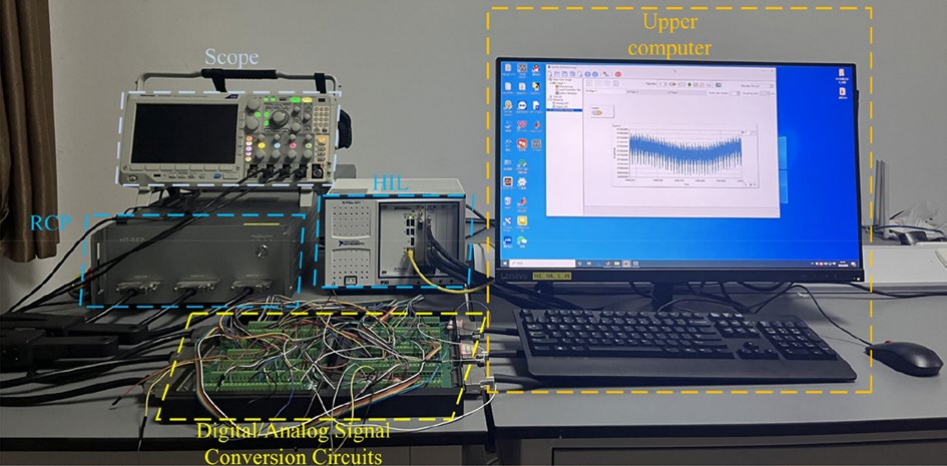
HIL + RCP experimental platform setup.

This section uses the HIL + RCP platform to experimentally validate the MPPT technology based on DBO (hardware in the loop, HIL; rapid control prototyping, RCP). StarSim HIL generates the analogue signal on field programmable gate array (FPGA) hardware and passes it to the controller. The digital/analogue signal interposer board enables signal connection between the simulation side and the control side. The development algorithm is downloaded to the StarSim RCP built-in controller, and the control signal is generated and transmitted to the simulation side. Finally, a closed-loop experimental system is formed. The parameters of the PV cells and the main circuit used are shown in Table 9. The experimental setup is shown in Fig. 28. The PV array is connected in the series form of 2 × 1 (a series/parallel configuration of 35 × 20 is used inside each PV cell), and the irradiance is 1000 W/m^2^ and 500 W/m^2^. The GM of the PV array is 39,864 W.

**Table 9:** Parameters of PV modules and other modules used for experimental verification.

Component	Value	Component	Value
Nominal maximum power (P_mp_)	53.07	Inductance (L)	1.1478e−3H
Optimal voltage (V_mp_)	17.4	Input capacitor (C_in_)	1e−3F
Optimal current (I_mp_)	3.05	Output capacitor (C_out_)	3227e−6F
Open-circuit voltage (V_oc_)	21.7	Conversion frequency (f)	10 kHz
Short-circuit current (I_sc_)	3.35	Load (RL)	100 Ω

**Figure 29:**
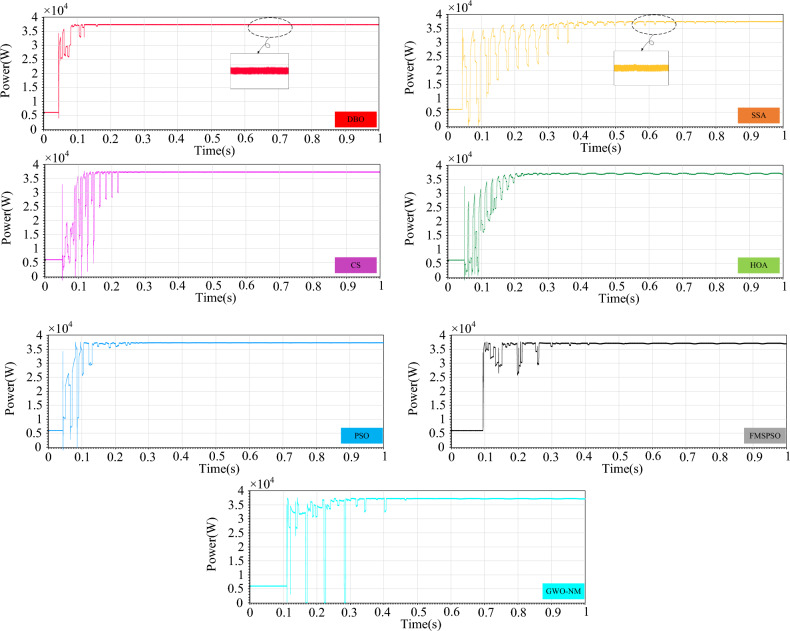
Comparison of power in the experiment.

**Figure 30:**
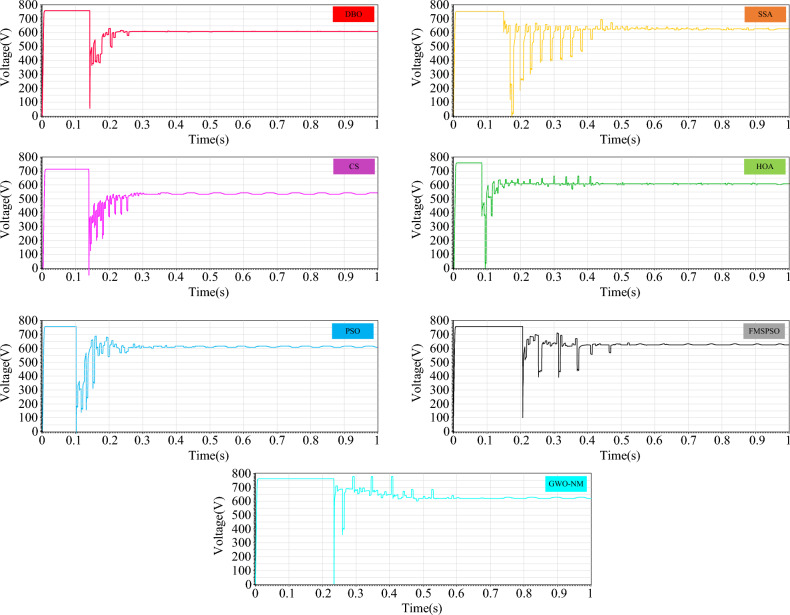
Comparison of voltage in the experiment.

Figure 29 shows the power comparison of five MPPT technologies in the online hardware closed-loop experiment. From the enlarged view, it can be seen that there is significant power oscillation in the HOA and SSA after reaching the GM. The powers tracked by DBO, SSA, CS, HOA, PSO, FMSPSO and GWO-NM are 37,387 W, 37,138 W, 37,320 W, 36,333 W, 37,207 W, 37,255 W, and 37,363 W, respectively, and the GM tracking efficiency of DBO reaches 93.4%. Figure 30 shows a comparison of the experimental voltages. The output voltages of the DBO, SSA, CS, HOA, PSO, FMSPSO and GWO-NM are 608.4 V, 632.5 V, 583.4 V, 606.5 V, 605.1 V, 641.3 V, and 644.5 V, respectively. It can be seen from the figure that the voltage of the DBO has no oscillation, while other MPPT technologies have different degrees of voltage fluctuation. Voltage fluctuations will eventually still lead to power loss, reducing the control efficiency of the MPPT. DBO also achieves efficient GM tracking on hardware systems with fast global and local convergence and zero power oscillation. This matches the results of the offline model run above.

## Conclusion

This study proposes a new MPPT technology based on DBO, aiming to solve the problems of power loss, GM oscillation and low tracking efficiency existing in traditional and intelligent MPPT technologies. By using different cases such as field atmospheric data, static/dynamic PSC and CPS in Shihezi city, a comparative analysis was conducted between DBO and renowned MPPT controllers such as SSA, CS, HOA, PSO, FMSPSO and GWO-NM proposed in recent years. The results show that DBO has excellent GMPP tracking capabilities under different weather conditions, and its GM tracking efficiency reaches 99.99% under all PSCs. Compared with other technologies, the convergence rate of DBO is increased by 38–80%, the stabilization rate is increased by 60–80%, and the energy harvest is increased by 3–8%. In the HIL + RCP hardware platform, the GM tracking efficiency of the DBO reached 93.4%, which is higher than other technologies, and the output power is more stable. Therefore, the new MPPT technology based on DBO can effectively solve the problems of random power oscillation, low tracking efficiency and easy falling into LM traps in PSCs and CPS. Because the DBO controller has the capabilities of fast convergence, efficient GM tracking and high energy capture, it is expected to improve the utilization efficiency of solar energy and the economic performance of PV systems. The PV system in this study is an off-grid type, and future research can apply the proposed algorithm to a grid-connected PV system to further test the performance of the proposed MPPT for practical applications.

## Data Availability

The datasets used or analyzed during the current study are available from the corresponding author on reasonable request.

## Abbreviations

PV: Photovoltaic
DBO: Dung beetle optimization
PSC: Partial shadow condition
CPS: Complex partial shadow
SSA: Squirrel search algorithm
CS: Cuckoo search optimization
HOA: Horse herd optimization
PSO: Particle swarm optimization
MPPT: Maximum power point tracking
FMSPSO: Adaptive factor selection multi-swarm PSO
GWO-NM: Hybrid Grey wolf optimizer-Nelder mead
GMPP: Global maximum power point
HIL: Hardware-in-loop
RCP: Rapid control prototype
LM: Local maxima
GM: Global maxima

## List of symbols

I: Output current
V: Output voltage
I_ph_: Photogenerated current
q: Electronic charge
R_ph_: Equivalent parallel resistance
R_sh_: Equivalent series resistance
k: Boltzmann's constant
T: Operating temperature
N_m_: Number of cells connected in series
N_p_: Number of cells connected in parallel
A: Characteristic constant
I_o_: Equivalent diode current
α: Natural coefficient
Lb^∗^: Lower bound of the spawning area
Ub^∗^: Upper bound of the spawning area
g: Random vectors
